# Clinical determinants of mortality in congenital diaphragmatic hernia: a single-center cohort study from Colombia

**DOI:** 10.3389/fped.2026.1907855

**Published:** 2026-08-05

**Authors:** Jorge Alvarado-Socarras, Juan Pablo Otoya-Castrillon, Claudia C. Colmenares-Mejía, Doris C. Quintero-Lesmes, Andrés Felipe Rubio-Duarte, Edgar Fabian Manrique-Hernandez, Andrea Candia, Angelica Ortiz-Cordoba, Juliana Ballesteros-Trillos, Alvaro Duran-Hernandez, Leonardo Salazar-Rojas

**Affiliations:** 1 Neonatal Intensive Care Unit, Hospital Internacional de Colombia HIC, Fundación Cardiovascular de Colombia FCV, Fundación Universitaria FCV, Santander, Colombia; 2 Pediatric Surgeon, Hospital Internacional de Colombia HIC, Fundación Cardiovascular de Colombia FCV, Fundación Universitaria FCV, Santander, Colombia; 3 Research Institute, Hospital Internacional de Colombia HIC, Fundación Cardiovascular de Colombia FCV, Fundación Universitaria FCV, Santander, Colombia; 4 Epidemiology and Data Analysis Unit, Hospital Internacional de Colombia HIC, Fundación Cardiovascular de Colombia FCV, Fundación Universitaria FCV, Santander, Colombia; 5 Pediatric Cardiac Intensive Care Unit, Hospital Internacional de Colombia HIC, Fundación Cardiovascular de Colombia FCV, Fundación Universitaria FCV, Santander, Colombia; 6 ECMO and Ventricular Assistance Program, Hospital Internacional de Colombia HIC, Fundación Cardiovascular de Colombia FCV, Fundación Universitaria FCV, Santander, Colombia

**Keywords:** Colombia, congenital, critical care, diaphragmatic, hernias, hospital mortality

## Abstract

**Introduction:**

Congenital diaphragmatic hernia (CDH) is a severe congenital anomaly associated with high neonatal morbidity and mortality. Evidence from low- and middle-income countries remains limited. This study aimed to identify clinical predictors of mortality and describe outcomes in neonates with CDH treated at a high-complexity center in Colombia.

**Methods:**

**R**etrospective cohort study including all neonates with confirmed CDH managed between May 2007 and February 2026. The main outcome was in-hospital mortality. Prenatal, neonatal, respiratory, surgical, and clinical management variables were analyzed. Multivariable Poisson regression was performed among patients undergoing surgical repair.

**Results:**

91 neonates were included, with an overall survival of 54.9% (95% CI 44.5–64.9). Prenatal diagnosis was established in 74.7% of cases, and 68.1% of patients achieved sufficient stabilization to undergo surgical repair. Non-survivors had higher rates of perinatal asphyxia and lower birth weight and gestational age. Patients undergoing surgical repair showed significantly better initial oxygenation and gas exchange parameters. Among surgically treated patients, mortality was associated with non-primary repair, larger defect size, absence of hernia sac, delayed abdominal closure, and postoperative complications. In multivariable analysis, perinatal asphyxia (RR 6.1, 95% CI 2.34–16.1), intrathoracic liver position (RR 5.1, 95% CI 1.37–19.1) and left ventricular dysfunction (RR 4.0 95% CI 1.07–14.9) were independently associated with mortality, while higher birth weight was protective.

**Conclusions:**

We noted high mortality in this cohort in Colombia. Early markers of cardiopulmonary severity and anatomical severity were strongly associated with mortality, highlighting the importance of prenatal risk stratification and early multidisciplinary stabilization.

## Introduction

Congenital diaphragmatic hernia (CDH) is a developmental anomaly of the diaphragm that allows abdominal contents—such as the intestines, spleen, and liver—to enter the thoracic cavity, thereby impeding the normal development of the lung parenchyma and vasculature. Its incidence is approximately 1 in 3,000 live births, but with variable clinical presentation, initially determined by the severity of pulmonary hypoplasia (1). In addition to the mechanical defect, there is theoretically prior cellular damage, which leads to erratic embryonic development, even before the herniation process (2).

CDH is not a simple condition and may be associated with other congenital or genetic malformations in 25–60% of cases, with the cardiovascular system being the most commonly affected, although other systems, such as the renal and gastrointestinal systems, may also be involved. Furthermore, a genetic cause can be detected in up to 57% of cases with multiple malformations, and there are clearly recognized syndromes in the context of CDH, such as trisomies 13–18, chromosomal deletions like isochromosome 12p (Pallister-Killian syndrome), and others such as Cornelia de Lange, Fryns, and CHARGE syndromes, among others. All these associations indicate a poor prognosis. Therefore, it is evident that this is not a simple condition, with variability in its presentation and outcomes. Although pulmonary hypoplasia was traditionally thought to be the primary determinant, it is now known that pulmonary hypertension and cardiac dysfunction are key factors in survival (3).

Mortality rates vary and depend on the experience of the centers, as well as certain strategies established within them, which may result in less lung injury, optimal timing of surgery, therapeutic approaches aimed at preoperative stabilization, and even the use of extracorporeal membrane oxygenation (ECMO) (4). Although there is no standardization of care, concentrating expertise and standardizing management could improve outcomes (5). There is also the challenge of the low prevalence of this condition, which limits the possibility of comparative studies, and many of the recommendations are based on expert opinion. Despite this, there are parameters for oxygenation, ventilation, and early ventilatory management that have been adopted as guidelines for the initial care of these patients (6).

Therefore, we decided to conduct a study to analyze the clinical factors associated with mortality and describe the outcomes in neonates with CDH treated at a high-complexity center in Colombia.

## Methods

### Study design and participants

A retrospective observational cohort study was conducted. The study took place at a high-complexity center in northeastern Colombia with experience in managing newborns with CDH who were treated between May 2007 and February 2026. The start of follow-up was defined as the time of birth for patients born at the institution and as the time of hospital admission for patients referred from other institutions. The end of follow-up corresponded to hospital discharge, defined as discharge or death during the initial hospitalization. The primary outcome was in-hospital mortality, defined as death during the initial hospitalization.

All newborns with a confirmed diagnosis of CDH treated at the institution during the study period were included. The diagnosis was established through imaging studies or intraoperative findings. Patients referred to another institution without sufficient information on clinical outcomes were excluded. Patients were identified through a review of institutional clinical records.

### Covariables

Potentially relevant clinical and perinatal variables were collected. These included maternal and prenatal characteristics such as maternal age, prenatal diagnosis, presence of polyhydramnios, use of fetal therapies, and performance of the EXIT procedure. Neonatal variables at birth were also recorded, including sex, gestational age, birth weight in grams, mode of delivery, and the presence of perinatal asphyxia. Anatomical and clinical characteristics of the condition were also included, such as the side of the hernia, the location of the liver (intra-abdominal or intrathoracic), the presence of associated congenital heart disease, and other malformations. Liver location was determined by imaging studies or intraoperative findings, while perinatal asphyxia was defined based on clinical criteria documented in the medical record. Ventricular function at the initial echocardiographic assessment was categorized as no dysfunction, right ventricular dysfunction, left ventricular dysfunction, or biventricular dysfunction. For multivariable analysis, left ventricular dysfunction was reclassified as a binary variable including isolated left ventricular dysfunction and biventricular dysfunction. Additionally, early respiratory and gas exchange parameters were collected at the time of the first clinical assessment, including oxygenation index (OI), arterial pCO₂, arterial pO₂, and pH. Variables related to clinical management were also recorded, including the performance of postnatal surgery, the use of extracorporeal membrane oxygenation (ECMO) support, and the presence of complications during hospitalization. ECMO support was initiated according to institutional multidisciplinary criteria based on refractory hypoxemia, severe pulmonary hypertension, and/or hemodynamic instability despite maximal medical management.

The data were obtained by reviewing electronic medical records and institutional databases through a standardized data collection process conducted by trained staff. The definitions of the variables were based on clinical criteria documented in the institutional medical records.

### Sample size

Given the low incidence of CDH, no formal *a priori* sample size calculation was performed. Instead, all consecutive neonates with confirmed CDH treated at the institution during the ∼19-year study period were included, an approach consistent with single-center cohorts of rare congenital anomalies, in which enrollment is constrained by disease incidence rather than by a pre-specified target. For the multivariable analysis restricted to patients undergoing surgical repair, the final model included four predictor variables estimated from complete data on 56 patients, among whom no more than 12 were non-survivors (the total number of deaths observed in the surgical repair subgroup, *n* = 62).

### Statistical analysis

A descriptive analysis of the baseline characteristics of the cohort was performed. Continuous variables were summarized as medians and interquartile ranges or means and standard deviations, depending on their distribution, while categorical variables were expressed as frequencies and percentages. For bivariate analysis, characteristics were compared between surviving and non-surviving patients using Student's t-test or the Mann–Whitney U test for continuous variables, and the chi-square test or Fisher's exact test for categorical variables, as appropriate.

To identify independent predictors of mortality, multivariable regression analysis was performed using a modified Poisson model with robust variance estimation, as proposed by Zou, to directly estimate adjusted risk ratios (7). Variables with clinical relevance and those with a p value <0.20 in the univariable analysis were considered candidates for inclusion. A backward elimination strategy was used to obtain the most parsimonious model. Adjusted risk ratios (RRs) and their corresponding 95% confidence intervals (95% CI) were reported. Model discrimination was assessed using the area under the receiver operating characteristic curve (AUC). Internal validation of the final model was performed using bootstrap resampling (1,000 replicates) to estimate optimism-corrected discrimination (8). All analyses were performed using Stata version 15.

The study was approved by the Institutional Ethics Committee of the Colombian Cardiovascular Foundation (No. 424, May 30, 2017) and was conducted in accordance with the principles of the Declaration of Helsinki and Resolution 8430 of 1993 issued by the Colombian Ministry of Health. Given the retrospective nature of the study, the requirement for informed consent was waived by the ethics committee.

## Results

A total of 91 neonates with a diagnosis of CDH were included. Overall survival was 54.9% (95% CI 44.5–64.9). No significant differences were observed in prenatal variables. Among neonatal variables, perinatal asphyxia was significantly more frequent in non-survivors (41.5% vs. 6.0%; *p* < 0.001). In contrast, survivors had higher birth weight and gestational age (*p* = 0.018 and *p* = 0.032, respectively). No significant differences were found in EXIT procedures, sex, mode of delivery, or comorbidities (Table 1).

**Table 1:** Baseline clinical and perinatal characteristics of the study population according to survival status.

Variable	Overall	Survivors	Non-survivors	*p*
	*N* = 91	*n* = 50	*n* = 41	
	N (%)	N (%)	N (%)	
Prenatal variables				
Maternal age*	27.2 (6.0)	27.1 (6.1)	27.5 (5.9)	0.746
Maternal medical history	18 (19.7)	10 (20.0)	8 (19.5)	0.954
Singleton pregnancy	86 (94.5)	47 (94.0)	39 (95.1)	0.815
Polyhydramnios	26 (28.6)	12 (24.0)	14 (34.1)	0.189
Prenatal diagnosis	68 (74.7)	36 (72.0)	32 (78.1)	0.509
Fetal therapy	10 (10.9)	3 (6.0)	7 (17.1)	0.093
Neonatal variables				
Institutional delivery	62 (68.1)	33 (66.0)	29 (70.7)	0.630
Antenatal corticosteroid exposure	14 (15.4)	5 (10.0)	9 (21.9)	0.116
Perinatal asphyxia	20 (21.9)	3 (6.0)	17 (41.5)	<0.001
Male sex	47 (51.6)	23 (46.0)	24 (58.5)	0.234
Birth weight, g	2947 (2600–3170)	2985 (2760–3220)	2765 (2350–3100)	0.018
Gestational age, weeks	38 (37–38.6)	38 (37–39)	37 (36–38)	0.032
Mode of delivery				0.075
Cesarean section	72 (79.1)	43 (86.0)	29 (70.7)	
Vaginal delivery	19 (20.9)	7 (14.0)	12 (29.3)	
Ventricular dysfunction				<0.001
No	53 (58.2)	39 (78.0)	14 (34.1)	
RV	4 (4.4)	3 (6.0)	1 (2.4)	
LV	6 (6.6)	1 (2.0)	5 (12.2)	
BV	15 (16.5)	3 (6.0)	12 (29.3)	
Missing data	13 (14.3)	4 (8.0)	9 (21.9)	
EXIT procedure	35 (38.5)	17 (34.0)	18 (43.9)	0.334
Liver position**				0.124
Intrathoracic	33 (53.3)	21 (42.0)	8 (66.7)	
Intra-abdominal	29 (46.7)	29 (58.0)	4 (33.3)	
Associated congenital heart disease	9 (9.9)	4 (8.0)	5 (12.2)	0.111
Underwent surgical repair	62 (68.1)	50 (100)	12 (29.3)	<0.001
Preoperative ECMO	13 (14.3)	6 (12.0)	7 (17.1)	0.491

* Mean (standard deviation). ** Information available for 79 patients. RV, Right ventricle; LV, Left ventricle; BV, Bi-ventricle.

Regarding surgical repair, it was performed in only 68.1% of patients. Surgical repair was strongly associated with survival (100% vs. 29.3%; *p* < 0.001).

**Figure 1:**
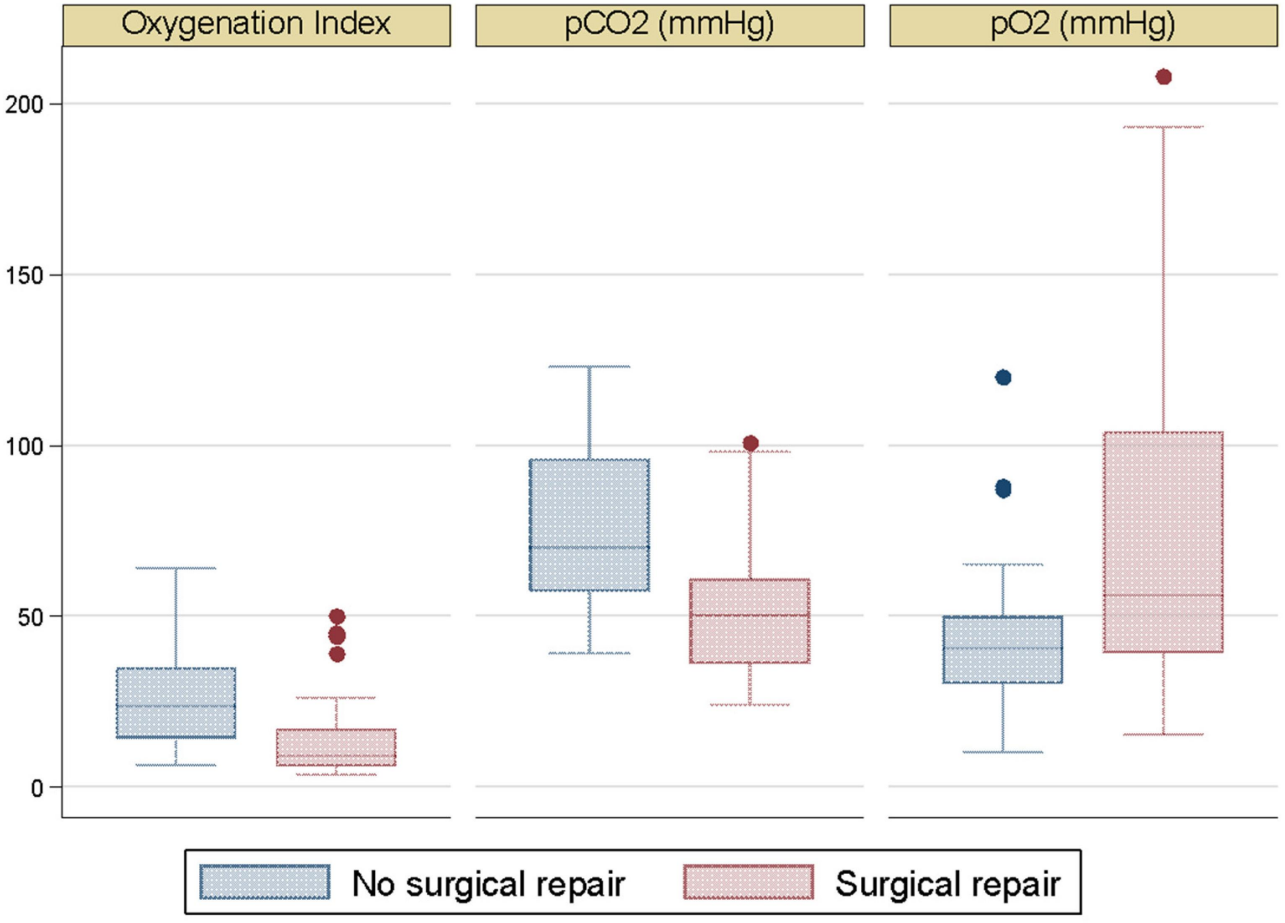
Initial oxygenation index, pCO₂, and pO₂ according to surgical repair status.

Information on initial oxygenation parameters was available for 68 patients (Table 2). Initial clinical parameters differed significantly according to surgical repair status. Patients who underwent surgical repair had significantly lower OI values compared with those who did not undergo surgery (median 9, IQR 6–17 vs. 23.5, IQR 14–35; *p* < 0.001). Similarly, initial pCO₂ levels were lower in the surgical repair group (50 mmHg, IQR 36–61 vs. 70 mmHg, IQR 57–96; *p* < 0.001). In contrast, patients who underwent surgical repair exhibited significantly higher pO₂ values compared with the non-surgical group (56 mmHg, IQR 39–104 vs. 40.5 mmHg, IQR 30–50; *p* < 0.001) (Figure 1).

**Table 2:** Clinical and surgical characteristics of patients undergoing surgical repair according to survival status.

Variable	Surgical repair	Survivors	Non-survivors	*p*
	*N* = 62	*n* = 50	*n* = 12	
	N (%)	N (%)	N (%)	
**Type of repair**				0.017
Primary	28 (45.2)	27 (54.0)	1 (8.3)	
Non-primary	31 (50.0)	21 (42.0)	10 (83.3)	
Missing data	3 (4.8)	2 (4.0)	1 (8.3)	
**Type of defect repaired**				0.007
A	1 (1.6)	1 (2.0)	1 (8.3)	
B	27 (43.5)	26 (52.0)	–	
C	20 (32.2)	15 (30.0)	1 (8.3)	
D	9 (14.5)	4 (8.0)	5 (41.6)	
Missing data	5 (8.1)	4 (8.0)	5 (41.6)	
**Hernia sac**				0.008
Yes	31 (50.0)	29 (58.0)	2 (16.7)	
No	22 (35.5)	13 (26.0)	9 (75.0)	
Missing data	9 (14.5)	8 (16.0)	1 (8.3)	
**Abdominal closure**				0.022
Primary	49 (79.0)	42 (84.0)	7 (58.3)	
Delayed	9 (14.4)	4 (8.0)	5 (41.7)	
Missing data	4 (6.4)	4 (8.0)	–	
Complications	26 (41.9)	17 (34.0)	9 (75.0)	0.010
ECMO rescue	17 (18.7)	6 (12.0)	11 (26.8)	0.071

**Figure 2:**
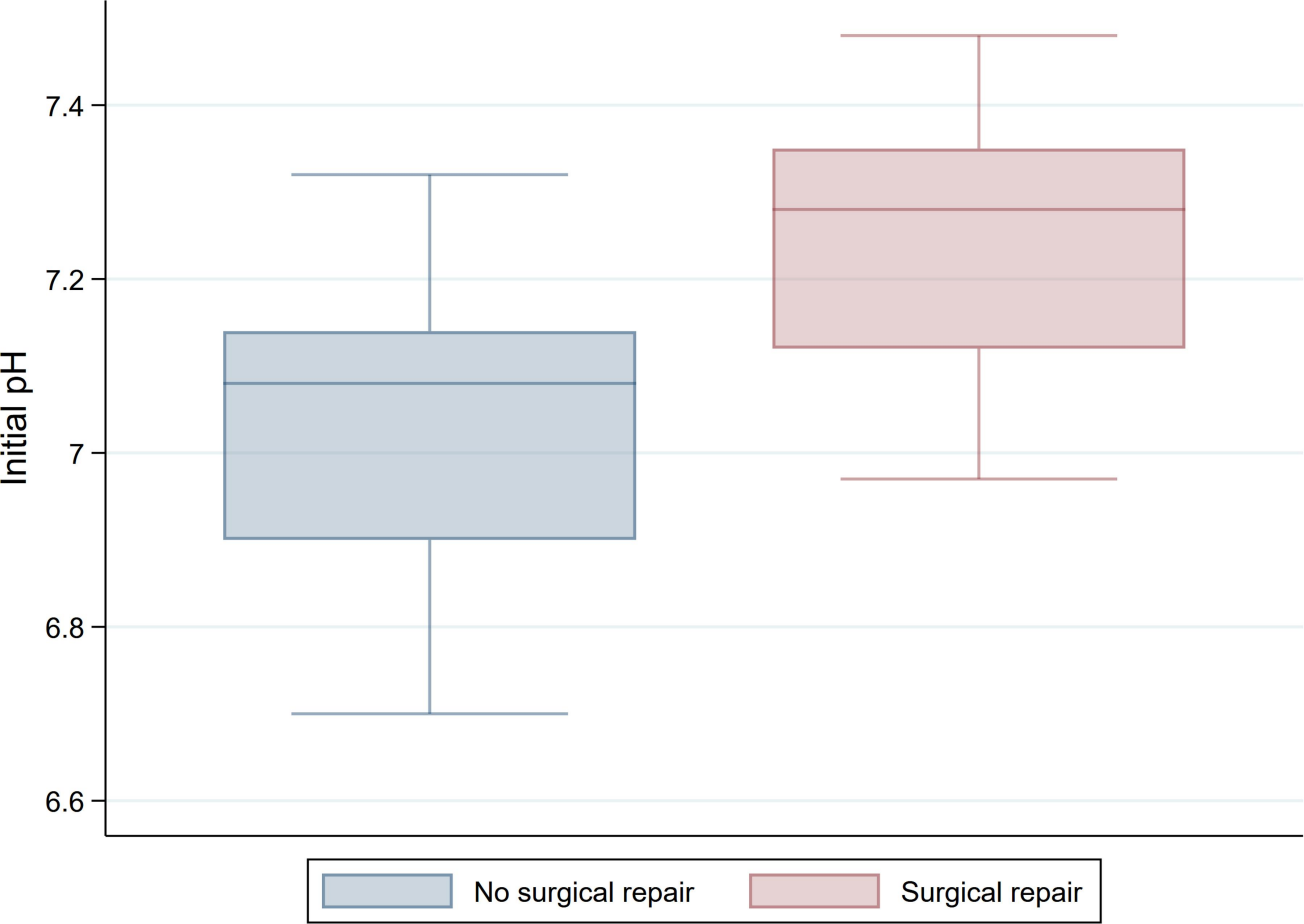
Initial pH according to surgical repair status.

Initial arterial pH also differed according to surgical repair status. Patients who underwent surgical repair had higher pH values compared with those who did not undergo surgery (median 7.28, IQR 7.12–7.35 vs. 7.08, IQR 6.90–7.14; *p* < 0.001) (Figure 2).

**Figure 3:**
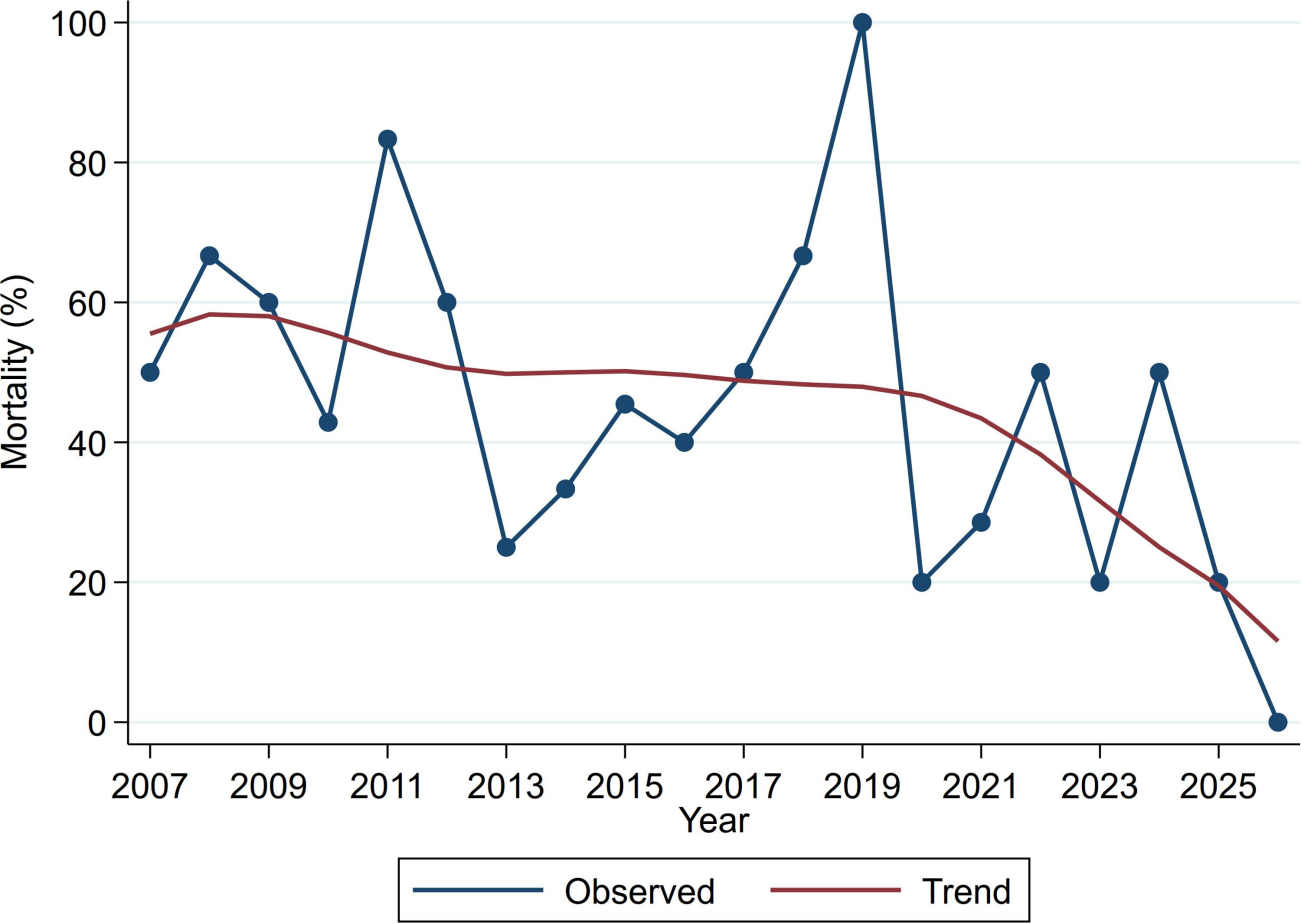
Temporal trend in mortality among patients with congenital diaphragmatic hernia.

Annual mortality showed substantial variability over time, with marked fluctuations between years. Despite this variability, the smoothed trend suggests a gradual decline in mortality rates in recent years. However, the wide year-to-year variation likely reflects small sample sizes within each period (Figure 3).

In the subgroup of neonates who underwent surgical repair (*n* = 62), differences were observed between survivors and non-survivors in variables related to defect severity and clinical course. Liver position did not show statistically significant differences (*p* = 0.124), although intrathoracic liver position was more frequent among non-survivors. Type of repair was associated with mortality (*p* = 0.017), with non-primary repairs being more common in non-survivors. Defect type was also significantly associated with mortality (*p* = 0.007), with the most severe defects (types C and D) being concentrated among non-survivors. Absence of a hernia sac was associated with higher mortality (*p* = 0.008). Delayed abdominal closure was more frequent among non-survivors (*p* = 0.022). Complications during hospitalization were significantly more common in this group (75.0% vs. 34.0%, *p* = 0.010). ECMO use was also more frequent among non-survivors, although this association did not reach statistical significance (*p* = 0.071). Initial ventricular dysfunction patterns also differed significantly according to survival status (*p* < 0.001). Biventricular dysfunction was markedly more frequent among non-survivors (29.3% vs. 6.0%), whereas absence of ventricular dysfunction was more commonly observed among survivors (78.0% vs. 34.1%). Left ventricular dysfunction was also more frequent in non-survivors (12.2% vs. 2.0%).

**Figure 4:**
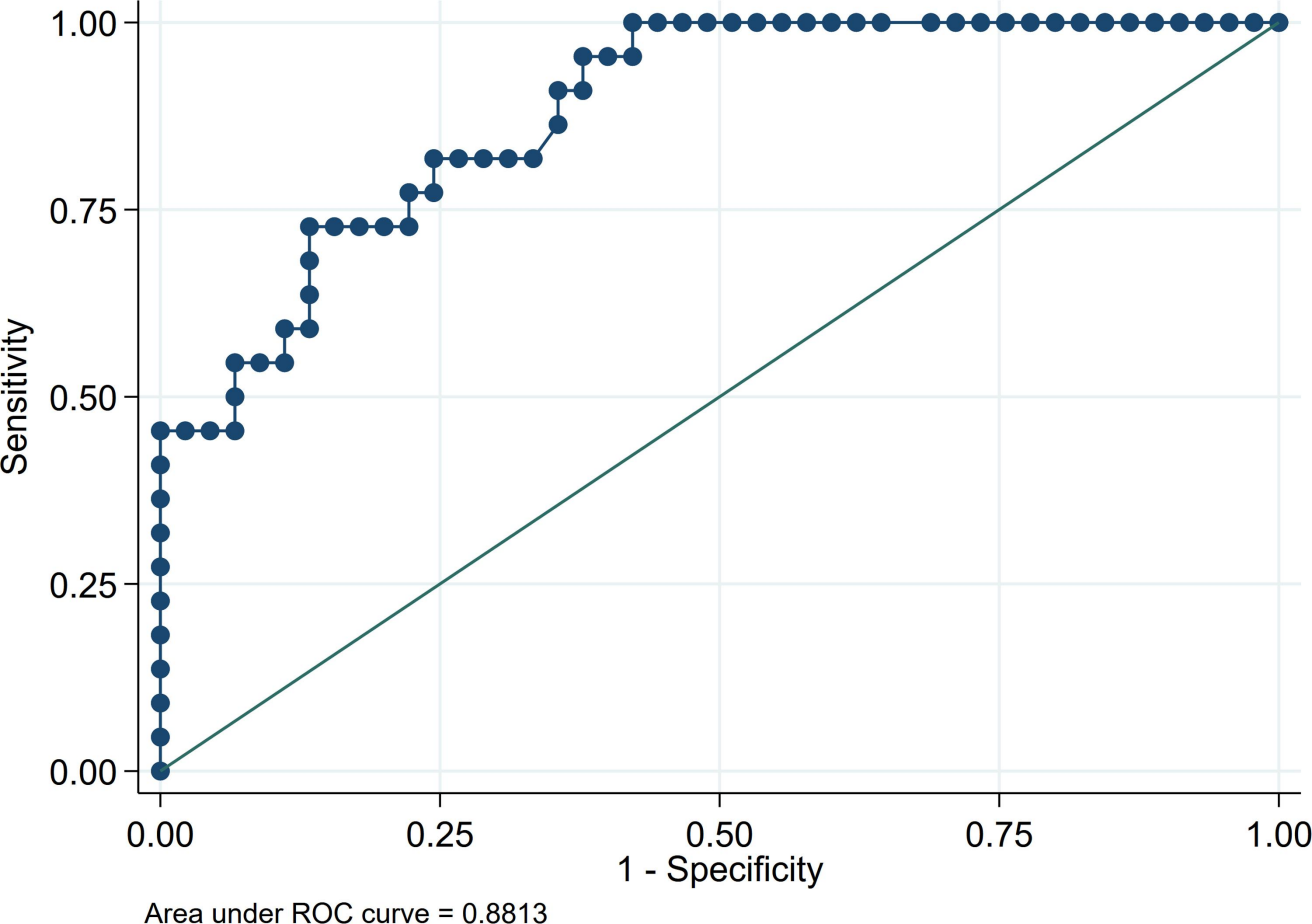
Receiver operating characteristic (ROC) curve for the multivariable model predicting mortality among patients undergoing surgical repair.

Among surgically treated patients, perinatal asphyxia (aRR 6.14, 95% CI 2.34–16.10), intrathoracic liver position (aRR 5.13, 95% CI 1.37–19.15), and left ventricular dysfunction (aRR 4.00, 95% CI 1.07–14.93) were independently associated with increased mortality, whereas higher birth weight was independently associated with a lower risk of mortality, with each additional 500 g associated with a 64% reduction in mortality risk (aRR 0.36, 95% CI 0.26–0.51) (Table 3). The final model was statistically significant (Wald *χ*^2^ = 33.08, *p* < 0.001) and demonstrated excellent discrimination, with an AUC of 0.88 (Figure 4). Bootstrap internal validation (1,000 resamples) indicated a mean optimism of 0.066, yielding an optimism-corrected AUC of 0.82 (2.5th–97.5th percentile range: 0.61–0.94), indicating that discrimination remained good after accounting for overfitting.

**Table 3:** Multivariable poisson regression model predictors of mortality among patients undergoing surgical repair .

Variable	aRR	95% CI	p-value
Perinatal asphyxia	6.14	2.34–16.10	<0.001
Birth weight (per 500 gram)	0.36	0.26–0.51	<0.001
Intrathoracic liver position	5.13	1.37–19.15	0.015
Left ventricular dysfunction	4.00	1.07–14.93	0.039

Left ventricular dysfunction included isolated left ventricular dysfunction and biventricular dysfunction. Complete-case multivariable analysis was performed in 56 patients with available echocardiographic and clinical data.

## Discussion

This study describes the clinical characteristics and outcomes of neonates with CDH treated at a high-complexity referral center in Colombia over an extended study period. Overall survival in our cohort was 54.9%, which remains below the outcomes reported by high-volume international centers and some Latin American ECMO centers, where survival rates between 70% and 80% have been described (9). Neonates with CDH experience a severely altered cardiopulmonary transition due to pulmonary hypoplasia and persistent pulmonary hypertension, resulting in poor cardiopulmonary adaptation that may lead to perinatal asphyxia and hypoxic-ischemic encephalopathy, both associated with high neonatal morbidity and mortality and the need for advanced support such as high-frequency ventilation, inhaled nitric oxide, or ECMO (10). However, our findings are comparable to reports from other middle-income countries, where persistent limitations in access to specialized perinatal care, delayed referral, heterogeneous prenatal follow-up, and variability in stabilization strategies continue to impact outcomes. These results highlight the challenges associated with managing severe CDH in resource-constrained settings despite the availability of advanced therapies such as ECMO (9, 11).

It is important to evaluate whether other associated factors, such as prematurity, congenital malformations, genetic syndromes, and even complications related to fetal therapy, may contribute to the proportion of patients who never achieve surgical eligibility. In CDH, not all newborns reach surgical repair because survival depends primarily on the degree of pulmonary hypoplasia and associated cardiopulmonary compromise. The presence of persistent pulmonary hypertension, refractory hypoxemia, hemodynamic instability (“no honeymoon”) (12), prematurity, or associated congenital malformations may prevent the clinical stabilization necessary to perform surgical repair (13, 14). Evaluation of these factors is essential to identify potentially avoidable contributors that may improve the likelihood of reaching surgery. In our cohort, survival among patients who underwent surgical repair was approximately 80%. Since perinatal asphyxia emerged as a major determinant of mortality, early cardiopulmonary adaptation appears to play a critical role in outcomes. Perinatal asphyxia was defined according to the diagnostic criteria in effect during each study period, acknowledging that temporal changes in clinical guidelines may have influenced the observed incidence and patient characteristics.

More broadly, the ∼19-year study period (2007– February 2026) encompassed substantial changes in the clinical management of CDH, including wider adoption of gentle ventilation and permissive hypercapnia strategies, a progressive shift toward preoperative stabilization with delayed elective repair rather than emergency surgery, increasing implementation of structured multidisciplinary protocols, and, in more recent years, greater availability of ECMO support and more consistent prenatal diagnosis. Although a formal before-after comparison was beyond the scope of this study, the observed temporal trend toward improved survival (Figure 3) is consistent with the cumulative effect of these evolving practices. However, because clinical criteria, diagnostic thresholds (including the definition of perinatal asphyxia), and treatment protocols were not uniform throughout the study period, temporal heterogeneity in management represents a potential source of unmeasured confounding that could not be fully accounted for in the multivariable model.

Current recommendations for initial management emphasize strategies aimed at reducing complications during the first 24 hours of life (4, 15, 16). More recent guidelines specifically adapted for low- and middle-income countries also emphasize clear oxygenation and ventilation targets to minimize pulmonary injury (17).

Data from our cohort also suggest a trend toward improved outcomes over time, which is consistent with findings from large referral centers where increasing experience, concentration of expertise, and implementation of institutional protocols have contributed to improved survival (4, 18). Interestingly, survivors had higher birth weight and gestational age. In addition, approximately 15% of patients had received antenatal corticosteroids, indirectly suggesting a risk of preterm delivery in a proportion of pregnancies. Therefore, optimal perinatal management aimed at prolonging pregnancy beyond 38 weeks whenever feasible may play an important role in improving outcomes. Current guidelines consistently identify prematurity as a major risk factor for mortality, even in high-income countries (19, 20).

Other important prognostic markers beyond perinatal variables were early blood gas parameters. Oxygenation index, pCO₂ levels, and initial pH clearly played a role in outcomes. Non-survivors exhibited higher OI values (reflecting more severe hypoxemia), higher pCO₂ levels (reflecting respiratory acidosis), and lower pH values (reflecting acidemia). Similar findings have been reported in other Latin American cohorts that also evaluated admission severity scores (21). Although mortality rates fluctuated during the study period, there appears to be a trend toward improved outcomes in recent years. This issue should continue to be evaluated from an interdisciplinary perspective given the persistently high mortality observed in low- and middle-income countries, including those in Latin America (11).

Several clinical markers were associated with mortality. One of the most important was intrathoracic liver position. Intrathoracic liver herniation (“liver-up”) is considered one of the most important prenatal predictors of poor prognosis in CDH because of its association with more severe pulmonary hypoplasia, severe pulmonary hypertension, and lower neonatal survival. Mullassery et al. demonstrated in a meta-analysis that fetuses with intrathoracic liver herniation had significantly lower survival compared with those with intra-abdominal liver position (45.4% vs. 73.9%), establishing this finding as an independent marker of disease severity (22). More recent reviews consistently continue to identify intrathoracic liver position as one of the strongest predictors of ECMO requirement and neonatal mortality in CDH patients (23). On the other hand, an additional marker of major clinical relevance is left ventricular dysfunction as an unfavorable prognostic factor (24). Our findings extend previous observations by showing that ventricular dysfunction remained independently associated with mortality even after accounting for other markers of disease severity. This supports the concept that myocardial performance represents an important component of the complex pathophysiology of CDH and should be considered during early risk assessment.

In the present study, the combination of perinatal asphyxia, birth weight, intrathoracic liver position, and left ventricular dysfunction demonstrated excellent discrimination for mortality (AUC 0.88). These variables likely reflect complementary dimensions of disease severity, including prenatal anatomical burden, cardiopulmonary adaptation at birth, overall physiological reserve, and myocardial performance. Although external validation is required, these findings suggest that integrating anatomical and functional markers may improve early risk stratification among infants who achieve surgical repair.

Among surgically treated patients, several intraoperative findings and surgical strategies were also associated with mortality. Larger defects (types C and D) clearly carry a higher risk of complications, including ECMO requirement, chronic lung disease, and death (25). Anatomical factors beyond defect size also appear to influence outcomes. The presence of a hernia sac is currently considered a favorable prognostic factor because it is associated with smaller diaphragmatic defects, lower ECMO requirement, and higher rates of primary closure (26). Several recent studies suggest that the presence of a hernia sac reflects less disruption of fetal thoracoabdominal development, allowing better preservation of abdominal domain and reducing the need for prosthetic patch repair and delayed abdominal closure (2). In contrast, large defects requiring prosthetic patch repair are associated with greater surgical morbidity, including recurrence, chylothorax, intestinal obstruction, and increased risk of intra-abdominal hypertension following visceral reduction (27). In these patients, delayed abdominal closure strategies using silos, temporary meshes, or progressive fascial closure are recommended to prevent abdominal compartment syndrome and postoperative respiratory deterioration. Consequently, contemporary guidelines emphasize the need to individualize surgical repair techniques according to defect size, closure tension, and neonatal abdominal capacity (16).

Regarding ECMO as rescue therapy, survival in our cohort was 35%, which remains below international standards reporting survival rates between 49% and 62% according to ELSO reports, with some evidence suggesting decreasing survival trends in recent years. The potential benefit of ECMO is primarily supported by observational studies suggesting improved outcomes in selected patients. However, these results are heavily influenced by patient selection criteria, which vary substantially among institutions and according to disease severity, including patients with absent “honeymoon” physiology, suggesting potentially irreversible cardiopulmonary compromise (28).

This study has several strengths. It represents one of the largest reported cohorts of CDH in Colombia and provides valuable data from a middle-income country, where evidence on CDH outcomes remains limited. The study includes a comprehensive evaluation of prenatal, neonatal, respiratory, surgical, and ECMO-related variables, allowing an integrated assessment of factors associated with mortality. In addition, the analysis of early gasometric parameters and the subgroup of surgically treated patients provides clinically relevant information regarding initial stabilization and surgical outcomes in this population. However, several limitations should be acknowledged. First, the retrospective single-center design depended on the quality and completeness of medical records, resulting in missing data for some prenatal and intraoperative variables. In addition, the ∼19-year study period was accompanied by evolving diagnostic criteria and management protocols, as discussed above, which may have introduced unmeasured temporal confounding despite the observed trend toward improved survival over time. Important prenatal severity markers such as observed-to-expected lung-to-head ratio (O/E LHR) were not consistently available throughout the study period. Although some patients were likely assessed prenatally, these data were not consistently documented in the medical records and therefore could not be reliably retrieved for analysis. As a result, adjustment for baseline prenatal disease severity was not possible, and residual confounding may have influenced the observed outcomes. Second, the subgroup analysis among surgically treated patients may be subject to selection bias, since only patients achieving sufficient cardiopulmonary stabilization underwent surgery. This form of selection, in which the sickest neonates die before reaching surgery, is expected to bias the multivariable model toward a survivor cohort with a narrower spectrum of disease severity, and the reported risk ratios should therefore be interpreted as prognostic factors among infants who achieve surgical eligibility rather than as predictors of mortality across the entire CDH population. Notably, perinatal asphyxia, low birth weight, and intrathoracic liver position were also associated with failure to reach surgery in the overall cohort (Table 1), suggesting that the direction of the bias, if anything, is likely to be conservative with respect to these markers, although this cannot be confirmed without data on patients who died before stabilization could be attempted. In addition, the model was internally evaluated but not externally validated, and therefore its predictive performance should be interpreted cautiously until confirmed in independent cohorts. Bootstrap internal validation indicated a mean optimism of 0.066, yielding an optimism-corrected AUC of 0.82 (2.5th–97.5th percentile range: 0.61–0.94), indicating that discrimination remained good after accounting for overfitting. Finally, the relatively small sample size limited the number of variables that could be included in the multivariable analysis. This constraint, together with the single-center design, underscores the need for multicenter or ambispective collaborations across Latin American CDH referral centers, which would increase sample size and statistical power, allow external validation of the present model, and improve the generalizability of findings to more heterogeneous settings.

These findings have important clinical implications for the management of CDH in middle-income settings. Early identification of markers associated with severe cardiopulmonary compromise, such as perinatal asphyxia, impaired gasometric parameters, and intrathoracic liver position, may facilitate timely risk stratification and optimize decision-making regarding stabilization, referral, and advanced therapies including ECMO. In addition, the observed trend toward improved survival over time highlights the potential impact of standardized management protocols, multidisciplinary care, and concentration of expertise in specialized referral centers. Strengthening prenatal diagnosis, delivery planning, and early postnatal stabilization strategies may contribute substantially to improving outcomes in neonates with CDH.

## Conclusions

CDH continues to be associated with high neonatal mortality in middle-income settings despite the availability of advanced therapies such as ECMO. In this cohort, mortality was strongly associated with markers of early cardiopulmonary instability and anatomical severity, particularly perinatal asphyxia, impaired initial gasometric parameters, low birth weight, intrathoracic liver position, and left ventricular dysfunction. Importantly, only a proportion of patients achieved sufficient stabilization to undergo surgical repair, highlighting the critical role of early multidisciplinary management during the neonatal transition period. These findings emphasize the importance of prenatal risk stratification, standardized stabilization protocols, and referral to specialized centers to improve outcomes in neonates with CDH.

## Data Availability

The datasets generated and analyzed during the current study are not publicly available due to ethical and institutional restrictions aimed at protecting patient confidentiality. Requests to access the datasets should be directed to claudiacolmenares@fcv.org.
